# Supplementary material for: Dopamine and Norepinephrine Tissue Levels in the Developing Limbic Brain Are Impacted by the Human CHRNA6 3′-UTR Single-Nucleotide Polymorphism (rs2304297) in Rats
Source: Int J Mol Sci. 2024 Mar 26;25(7):3676. doi: 10.3390/ijms25073676 (PMC11011259; doi:10.3390/ijms25073676)
Supplement: Supplementary file 1 [file ijms-25-03676-s001.zip › ijms-2908897-supplementary.pdf]

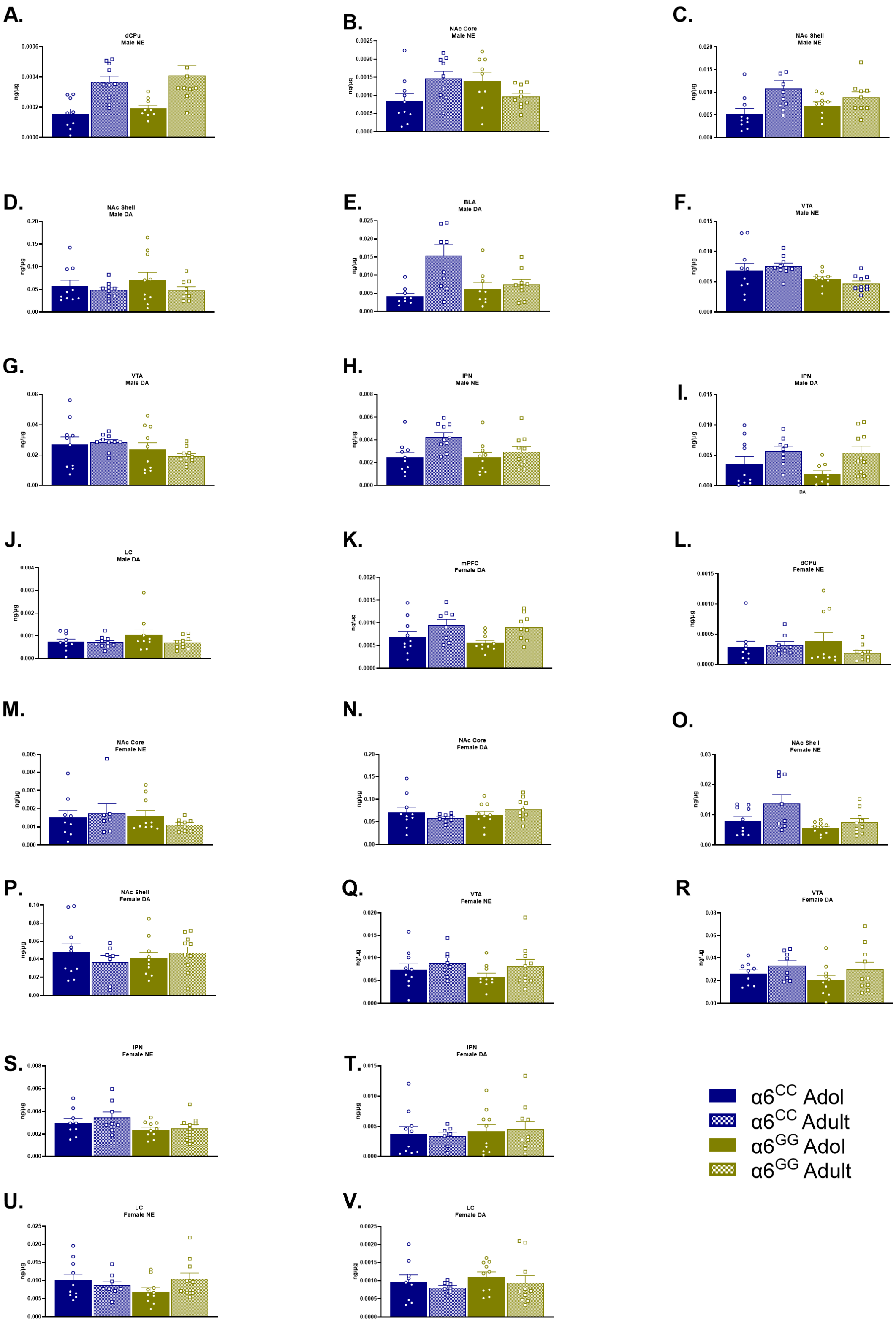

Supplemental Figure S1 **DA and NE in human *CHRNA6* 3'-UTR SNP knock-in in regions of the reward system. DA and NE** Means ± SEM in males (A-J) and females (H-V). dCPu = dorsal Caudate putamen, NAc = Nucleus accumbens (Shell and Core), VTA (Ventral tegmental area, IPN = Interpeduncular nucleus, LC = Locus coeruleus $\alpha6^{GG}$ vs $\alpha6^{CC}$ All data presented as mean ± SEM. N=8-10/group.

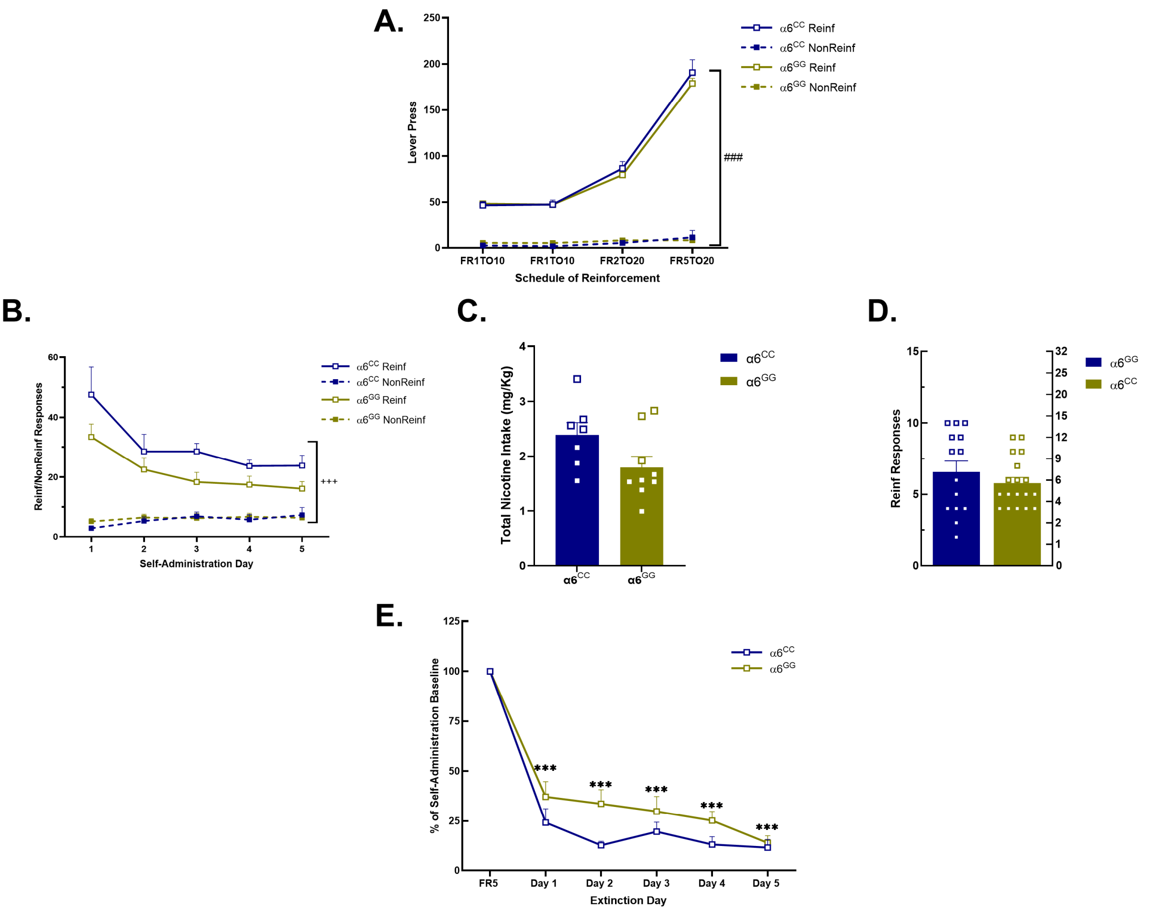

Supplemental Figure S2 The human *CHRNA*6 3'-UTR SNP knock-in does not impact food self-administration, nicotine self-administration, nicotine intake, progressive ratio, and extinction in males. (A) Males, $\alpha6^{GG}$ and $\alpha6^{CC}$, mean daily 30 min responses ± SEM for food self-administration at Fixed Ratio (FR)1 Time out (TO)1, FR1TO10, FR2TO20, and FR5TO20 schedules of reinforcement. ###p<0.0001 Reinforced (Rein) vs. Non-Reinforced (NonReinf) responses. N=7-9/group. (B) Males, $\alpha6^{GG}$ and $\alpha6^{CC}$, mean daily 1-h responses ± SEM for nicotine self-administration at FR5TO20 schedule of reinforcement. ***p<0.001 Rein vs. NonReinf. N=7-9/group. (C) Males, $\alpha6^{GG}$ and $\alpha6^{CC}$, total nicotine intake and (D) progressive ratio. (E) After completion of nicotine self-administration, males, $\alpha6^{GG}$ and $\alpha6^{CC}$, were allowed to respond to the Rein and NonReinf lever without schedule consequence (e.g. infusion of nicotine, cue light and tone). Data are presented as a mean ± SEM percent of the last day of nicotine self-administration responding for the first five days. ***p<0.001 vs Last FR5. N=7-9/group.

Supplemental Table S1 Summary of NE and DA in CHRNA6 3'-UTR SNP Knock-In Adolescent and Adult, Male and Female, Key Regions of the Reward Circuitry

		**Females**				**Males**			
		$\alpha 6^{CC}$		$\alpha 6^{GG}$		$\alpha 6^{CC}$		$\alpha 6^{GG}$	
		Adolescent	Adult	Adolescent	Adult	Adolescent	Adult	Adolescent	Adult
PFC	DA						↑	↑	
	NE	↑			↑		↑	↑	
CPu	DA						↑		
	NE				↑				
NAc Core	DA								
	NE						↑	↑	
BLA	DA				↑		↑		
	NE				↑				
LC	DA							↑	
	NE							↑	

Prefrontal cortex (PFC); Caudate Putamen (CPu); Nucleus Accumbens (NAc); Basolateral Amygdala (BLA); Locus Coeruleus (LC); Dopamine (DA); Norepinephrine (NE)

Supplemental Table S2 DOPAC/DA and HVA/DA turnover in central regions of the reward system for naïve adolescent, adult and drug-seeking rats with the *CHRNA6* 3'UTR SNP.

		α6^{CC}			α6^{GG}		
		Adolescent	Adult	Reinstatement	Adolescent	Adult	Reinstatement
mPFC	DOPAC/DA	1.14 ± 0.27	1.35 ± 0.25	2.15 ± 0.28	1.02 ± 0.25	0.46 ± 0.27	0.99 ± 0.19
	HVA/DA	0.63 ± 0.13	0.45 ± 0.12	3.81 ± 1.57**++	0.65 ± 0.13	0.6 ± 0.12	2.86 ± 1.06*+
dCPu	DOPAC/DA	0.24 ± 0.02	0.16 ± 0.02	0.17 ± 0.01*	0.18 ± 0.02	0.14 ± 0.02	0.14 ± 0.01
	HVA/DA	0.05 ± 0.01	0.07 ± 0.01	0.03 ± 0.01++	0.04 ± 0.01	0.07 ± 0.01	0.04 ± 0.00++
NAc Core	DOPAC/DA	0.33 ± 0.02	0.28 ± 0.02	0.29 ± 0.03	0.2 ± 0.02	0.24 ± 0.02	0.25 ± 0.02
	HVA/DA	0.04 ± 0.01	0.09 ± 0.01	0.07 ± 0.01	0.03 ± 0.01	0.11 ± 0.01	0.06 ± 0.01++
NAc Shell	DOPAC/DA	0.2 ± 0.02	0.15 ± 0.02	0.27 ± 0.03+++	0.16 ± 0.02	0.14 ± 0.02	0.23 ± 0.02*++
	HVA/DA	0.03 ± 0.01	0.04 ± 0.01	0.15 ± 0.11	0.05 ± 0.01	0.04 ± 0.01	0.29 ± 0.07***+++
BLA	DOPAC/DA	0.39 ± 0.04	0.15 ± 0.04	0.29 ± 0.06	0.26 ± 0.04	0.14 ± 0.04	0.21 ± 0.04
	HVA/DA	0.14 ± 0.03	0.06 ± 0.03	0.31 ± 0.15	0.14 ± 0.03	0.1 ± 0.03	0.35 ± 0.10++
VTA	DOPAC/DA	0.39 ± 0.03	0.31 ± 0.03	0.24 ± 0.09	0.35 ± 0.03	0.28 ± 0.03	0.22 ± 0.06*
	HVA/DA	0.08 ± 0.02	0.1 ± 0.02	0.3 ± 0.33	0.13 ± 0.02	0.11 ± 0.02	0.62 ± 0.20***+++
IPN	DOPAC/DA	1.27 ± 0.18	0.63 ± 0.19	12.95 ± 3.53***+++	0.95 ± 0.19	0.48 ± 0.18	2.57 ± 1.66
	HVA/DA	1.22 ± 0.32	0.25 ± 0.33	5.27 ± 1.82**+++	1.06 ± 0.32	0.38 ± 0.32	2.24 ± 0.86+
LC	DOPAC/DA	1.47 ± 0.54	3.89 ± 0.51	2.45 ± 1.50	1.17 ± 0.51	1.72 ± 0.54	3.16 ± 0.92
	HVA/DA	1.08 ± 0.40	0.56 ± 0.40	5.27 ± 3.17+	1.16 ± 0.40	0.91 ± 0.40	6.86 ± 1.94***+++

Prefrontal cortex (PFC); Caudate Putamen (CPu); Nucleus Accumbens (NAc); Basolateral Amygdala (BLA); Locus Coeruleus (LC); Dopamine (DA); Norepinephrine (NE); 3,4-Dihydroxyphenylacetic acid (DOPAC); Homovanillic acid (HVA)

*p<0.05, **p<0.01, ***p<0.001 vs naïve adolescents; +p<0.05, ++p<0.01, +++p<0.001 vs naïve adults.
